# Redox Imbalance and Viral Infections in Neurodegenerative Diseases

**DOI:** 10.1155/2016/6547248

**Published:** 2016-03-27

**Authors:** Dolores Limongi, Sara Baldelli

**Affiliations:** Università Telematica San Raffaele Roma, Via di Val Cannuta, 00167 Rome, Italy

## Abstract

Reactive oxygen species (ROS) are essential molecules for many physiological functions and act as second messengers in a large variety of tissues. An imbalance in the production and elimination of ROS is associated with human diseases including neurodegenerative disorders. In the last years the notion that neurodegenerative diseases are accompanied by chronic viral infections, which may result in an increase of neurodegenerative diseases progression, emerged. It is known in literature that enhanced viral infection risk, observed during neurodegeneration, is partly due to the increase of ROS accumulation in brain cells. However, the molecular mechanisms of viral infection, occurring during the progression of neurodegeneration, remain unclear. In this review, we discuss the recent knowledge regarding the role of influenza, herpes simplex virus type-1, and retroviruses infection in ROS/RNS-mediated Parkinson's disease (PD), Alzheimer's disease (AD), and amyotrophic lateral sclerosis (ALS).

## 1. Introduction

Neurodegenerative diseases are chronic degenerative pathologies of the Central Nervous System (CNS) characterized by progressive loss of specific neurons that lead to a decline in brain functions [1–3]. Despite these pathologies having different clinical features, they possess some common hallmarks, such as the formation and deposition of aberrant protein conformers, synaptic dysfunctions, deficient autophagic processes, oxidative/nitrosative stress, and inflammation [4]. The neurodegenerative diseases present an increase of reactive oxygen species (ROS) production by mitochondria and NADPH oxidase (NOX), which seems to be responsible for tissue injury, inflammation, and neurodegeneration [5, 6].

Substantial evidence indicates that also reactive nitrogen species (RNS) play a key role in most common neurodegenerative diseases although the mechanism of nitric oxide- (NO-) mediated neurodegeneration remains uncertain [7–9]. However, many studies demonstrated that NO is able to modify protein function by nitrosylation and nitrotyrosination, contribute to glutamate excitotoxicity, inhibit mitochondrial respiratory complexes, participate in organelle fragmentation, and mobilize zinc from internal stores in brain cells, contributing to neurodegeneration [10–13]. In response to increased oxidative and nitrosative stress the brain cells (i.e., microglia, astrocytes) activate redox-sensitive transcription factors, including nuclear factor-k*β* (NF-k*β*) and activator protein-1 (AP-1) [14, 15]. Next to this, it was also observed that the free radical increase, observed during neurodegeneration, may be also due to alteration of endogenous antioxidants. In particular, some antioxidant enzymes, such as superoxide dismutases (SODs), catalase, glutathione peroxidase, and glutathione reductase, have reduced activity in certain brain regions of AD patients [16]. Moreover, a reduction in amount of glutathione (GSH) level has been found in postmortem brain tissue from the* substantia nigra* of PD patients [17, 18]. Similarly, catalase and glutathione reductase activity, as well as GSH levels, were found to be significantly reduced in ALS patients [19]. Many of these antioxidant systems are regulated by nuclear factor (erythroid-derived 2)-like 2, also known as NFE2L2 transcription factor. In normal conditions, NFE2L2 is associated with Kelch-like ECH associating protein 1 (Keap1) in the cytoplasm. This bond prevents the nuclear translocation of NFE2L2 and promotes its degradation* via* Ubiquitin Proteasome System (UPS). On the contrary, the presence of oxidative stress can induce the detachment between Keap1 and NFE2L2, due to the modification of the reactive cysteine in Keap1 [20]. These conformational changes determine a release of NFE2L2 and its nuclear translocation, where it binds the ARE consensus sequences and coordinates the transcription of antioxidant and phase II detoxification genes [21]. Alterations of NFE2L2-pathway have been observed in postmortem brain of patients with neurodegenerative disorders [20]. In particular, many studies have showed an increase of NFE2L2 nuclear translocation in dopaminergic neurons of PD patients, but this induction is not sufficient to counteract the oxidative stress [22]. On the contrary, a decrease of NFE2L2 expression has been observed in hippocampus neurons in AD cases [22]. Moreover, a reduction of mRNA and protein levels of NFE2L2 was also found in the motor cortex and spinal cord in ALS patients [23]. Thus, the activation of NFE2L2-ARE pathway constitutes a valuable therapeutic tool to combat oxidative stress that occurs during neurodegenerative disease.

Recently, it has been demonstrated that infection agents can reach the CNS crossing the blood-brain barrier, by infected migratory macrophage or by intraneuronal transfer from peripheral nerves [24, 25]. In particular, these infections can affect the immune system resulting in a variety of systemic signs and symptoms [26]. The virus replication into the CNS produces molecular hallmarks of neurodegeneration, such as protein misfolding, deposition of misfolded protein aggregates, alterations of autophagic pathways, oxidative stress, neuronal functional alterations, and apoptotic cell death [26–28]. These effects associated with genetic alteration and other environmental factors contribute to the pathogenesis of neurodegenerative diseases.

In this review, we will highlight the role of oxidative stress and viral infection in the pathogenesis of Parkinson's disease (PD), Alzheimer's disease (AD), and amyotrophic lateral sclerosis (ALS).

## 2. Role of Oxidative Stress in Neurodegeneration: General Aspects

Oxidative stress occurs due to an imbalance in the prooxidant and antioxidant levels. ROS and RNS are highly reactive with biomolecules, including proteins, lipids, carbohydrate, DNA, and RNA [29]. ROS that are particularly abundant during an imbalance of redox state are superoxide anion (O_2_^∙−^), hydrogen peroxide (H_2_O_2_), and hydroxyl radical (^∙^OH), whereas among RNS the most abundant are NO and peroxynitrite (ONOO^−^). During mitochondrial activity O_2_^∙−^ is produced in the electron transport chain (ETC), which is immediately converted to H_2_O_2_ by superoxide dismutase 2 (SOD2) located in the mitochondrial matrix or SOD1 located in the cytosol [30]. H_2_O_2_ is rapidly converted to water by mitochondrial glutathione (mtGSH) with the participation of GSH reductase and peroxiredoxins [31]. Other sources of free radical are the NOXs, enzymes located in the cell membrane. Several NOXs are expressed in the cells of CNS, such as neurons, astrocytes, and microglia [32, 33]. During infections, activation of NOXs is strongly improved and the resulting ROS increase is particularly important as a host defense mechanism [34]. However, excessive NOXs activation has also been implicated in oxidative stress-mediated neurodegeneration [35].

**Figure 1:**
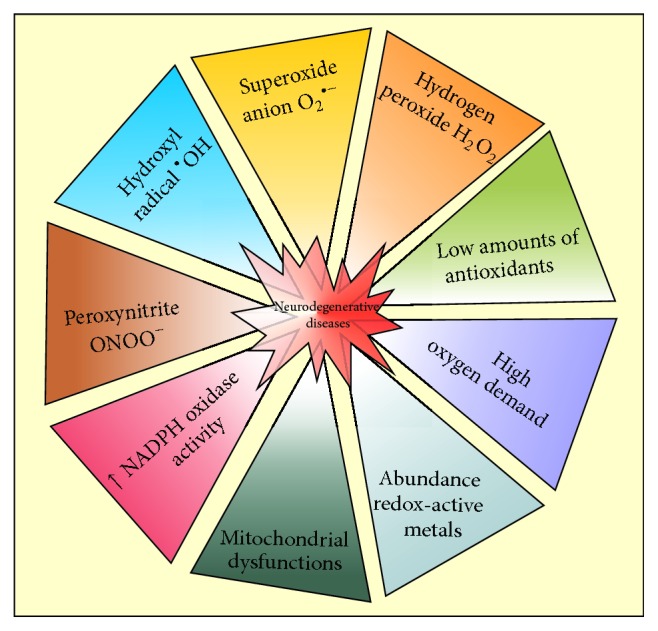
Main characteristics that occur in neurodegenerative diseases.

The brain is particularly prone to oxidative stress-induced damage because of its high oxygen demand, the abundance of redox-active metals (iron and copper), the high levels of oxidizable polyunsaturated fatty acids, and the low amounts of antioxidant enzymes (Figure 1). Another issue is that the neurons are postmitotic cells with relatively restricted replenishment by progenitor cells during the lifespan of an organism [11, 36]. Thus, the brain may be particularly vulnerable to viral infections during neurodegeneration due to different reasons: (i) the blood-barrier is compromised during neurodegeneration; (ii) many viruses can reach the CNS by peripheral nerves; (iii) the mitochondria become dysfunctional during neurodegeneration, preventing neurons from depending on aerobic metabolism and making it very susceptible to oxidative stress [17]. Primarily, in this review, the role of redox imbalance and redox-mediated inflammation in the onset and pathogenesis of neurodegenerative diseases will be discussed.

### 2.1. Redox Imbalance in AD

AD is a neurodegenerative disorder characterized by progressive decline in cognitive functions leading to memory loss and dementia. It involves degeneration of limbic and cortical brain structures, especially in the temporal lobe. One characteristic of AD is the appearance of senile plaques, which are produced from proteolytic cleavage of the transmembrane amyloid precursor protein (APP) to form *β*-amyloid peptide (A*β*). Another characteristic of AD is neurofibrillary tangles (NFTs) [37] and aggregates of medium and high molecular weight neurofilaments (NFM and NFH, resp.), as well as the microtubule-stabilizing protein tau, a multifunctional protein involved in microtubule assembly and stabilization [38, 39]. These hallmarks are altered in ways characteristic of oxidative damage, such as advanced glycation end product- (AGE-) modifications, protein cross-linking, and carbonyl-modifications [40–42]. All these alterations in neurons susceptible to AD play a key role in the irreversible cellular dysfunction that ultimately leads to neuronal death.

Brain autopsy from AD patients has shown oxidative damage markers, such as lipid peroxidation, protein oxidative damage, and glycoxidation in brain tissues [43]. Next to this, a drastic decrease in the intraneuronal content of GSH has been observed in the hippocampus and cortex of AD patients [43, 44]. Thus, the loss of ROS balance produces a chronic oxidative state, which induces a reduction of antioxidants expression and activity, accelerating the neurodegenerative processes. In fact, the alteration of redox homeostasis stimulates the formation of products of advanced glycosylation, an overload of peroxidation of fatty acids, oxidation of cholesterol, insulin resistance, and proteins unfolding [41, 45–49]. Moreover, an increase of Heme Oxygenase-1 (HO-1) and 8-hydroxyguanosine (8-OHG) was found in AD brain as compared with controls [50].

Despite the cause of redox imbalance still being unclear in AD pathogenesis, many studies suggest that the alteration in redox transition metals balance (i.e., iron, copper) is the major cause of neurodegeneration [51–53]. In fact, iron and copper have been found in high concentrations in AD brain. In particular, Zn, Cu, and Fe in senile plaques rims and cores have been found significantly elevated in AD [51]. It has also been demonstrated that the activity of many proteins, such as ferritin and ceruloplasmin, which are important to regulation of metal homeostasis, shows altered expression in AD [54]. Other studies have revealed dramatic drops in the levels of some biometals in the AD brain, which may aid development of senile plaques [55]. In particular, reduced levels of intracellular Cu have been reported in cortical neurons derived from AD transgenic mice and in the most-affected brain region of AD patients [56]. This alteration appears to contribute in part to AD pathogenesis. The dysregulation of biometal homeostasis in AD is a complex pathway, which has contributed to the development of new therapeutic approaches to restore the neuronal functions.

The combination of all these factors could explain how the oxidative stress is linked to the formation of amyloid plaques and NFTs in AD.

### 2.2. Redox Imbalance in PD

PD is progressive neurodegenerative disease characterized by extrapyramidal movement disorders that manifest as rigidity, resting tremor, and postural instability [57]. PD is also characterized by a progressive loss of dopaminergic neurons in the* substantia nigra*, accompanied by the accumulation of *α*-synuclein aggregates in Lewy bodies [58]. Lewy bodies are composed not only of *α*-synuclein, but also of other proteins, such as ubiquitin and neurofilament proteins [59].

Many evidences demonstrate that oxidative stress plays an important role in PD pathogenesis. The* substantiae nigrae* of PD subjects show increased levels of oxidized protein lipid [60], DNA [61], and decreased level of GSH [62]. In particular, oxidized proteins may not be adequately ubiquitinated and recognized by proteasome and thus accumulate within the neurons [63]. Moreover, DNA damage could determine an alteration of many important genes essential for neurons activity and functionality [64]. Increased levels of 4-hydroxynonenal (HNE) were found in the rime of Lewy bodies of PD [65]. HNE, activating caspase-8, caspase-9, and caspase-3 and inducing DNA fragmentation, is able to ultimately provoke apoptosis of dopaminergic cells [66]. HNE inhibits NF-k*β* pathway [67], induces PARP cleavage [68], decrease GSH content, and inhibits complexes I and II of the ETC, contributing to the disease progression [69–71].

Mice treated with PD toxins (i.e., 1-methyl-4-phenyl-1,2,3,6-tetrahydropyridine (MPTP), paraquat, and rotenone) support the link between oxidative stress and dopaminergic neuronal degeneration. In particular, MPTP causes a depletion of dopamine (DA) levels [72] and reduction of tyrosine hydroxylase (TH) [73]. The monoamine oxidase B (MO B) converts MPTP in 1-methyl-4-phenylpyridinium (MPP+), which blocks mitochondrial complex I and causes ATP depletion and ROS increase. This is thought to be the main cause of MPTP-induced terminal degeneration [74–77]. Consequently, MPTP-treated mice show an induction of glial response and increased levels of inflammatory cytokines and microglial activation, suggesting that the neurodegenerative process is evolving [78].

In the last years, the discovery of genes implicated to familial forms of PD (i.e., *α*-synuclein, Parkin, and DJ-1) has allowed the identification of new mechanisms, which highlight the importance of oxidative stress in PD pathogenesis. For example, *α*-synuclein gene mutations are linked with inherited PD and increase the tendency of the protein to aggregate [79]. It is a natively unfolded protein that can associate with vesicular and membranous structures and plays a role in synaptic vesicle recycling storage. Fibrils of *α*-synuclein in conjunction with DA were found in* substantia nigra*, which lead to an accumulation of cytotoxic soluble protofibrils and an increase of oxidative/nitrosative stress [80, 81].

### 2.3. Redox Imbalance in ALS

ALS is a relentlessly progressive neurodegenerative disorder, in which increasing muscle weakness leads to respiratory failure and death, which typically develops during the sixth or seventh decade of life [82].

Different studies show an increase of oxidative damage to proteins in ALS postmortem tissues compared to control. In particular, high levels of protein carbonyls have been identified in both spinal cord [83] and motor cortex [84] from ALS cases. Increased 3-nitrotyrosine levels were observed in both sporadic and SOD1 familial ALS patients [85]. Oxidative damage to DNA, measured by levels of 8-OHG, has also been found to be increased in cervical spinal cord from ALS patients [86]. Immunoreactivity to the brain and endothelial forms of nitric oxide synthase (eNOS) was also elevated in ALS motor neurons relative to controls, suggesting that nitration of protein-tyrosine residue is upregulated in motor neurons of the spinal cord of ALS [87].

Transgenic mouse models and cell culture models of ALS based on mutant SOD1 recapitulate the oxidative damage to protein, lipid, and DNA observed in the human disease [88]. Moreover, many studies have suggested that SOD1 mutations could have toxic effects for three different reasons: (i) loss of function leading to increased levels of O_2_^∙−^, which can react with NO to produce ONOO^−^ [85]; (ii) a dominant-negative mechanism whereby the mutant SOD1 protein not only is inactive, but also inhibits the function of normal SOD1 expressed by the normal allele [89]; or (iii) increased SOD1 activity leading to increased H_2_O_2_ levels and ^∙^OH [89].

A new pathological feature identified in postmortem tissue of ALS patients consists in neuronal protein deposition of TDP-43 or TAR DNA binding protein with a molecular mass of 43 kDa [90]. In particular, TDP-43 aggregates were found in 97% of ALS cases whether sporadic or familial [91, 92]. TDP-43 is a ubiquitously expressed DNA/RNA-binding protein, which is expressed in cytoplasm and in the nucleus where it regulates RNA splicing and microRNA biogenesis [93–95]. It has been observed that in conditions of oxidative stress TDP-43 is able to translocate in cytoplasm and assemble into stress granules (SGs), which are evident in ALS [96, 97]. SGs are large messenger ribonucleoprotein aggregates that are implicated in the stress-mediated inhibition of mRNA and protein synthesis [98]. An altered control of mRNA translation in stressful conditions may trigger motor neuron degeneration at early stages of the disease. Thus, the presence of TDP-43 in SGs leads to a loss of protein functionality defining an altered control of mRNA translation in stressful conditions triggering neuron degeneration.

## 3. Redox-Mediated Inflammation in Neurodegenerative Diseases

Recent studies have highlighted the correlation between oxidative damage and* neuroinflammation* in neurodegenerative processes, with the term neuroinflammation meaning the chronic inflammation of the CNS. It is characterized by inflammatory molecules expression, endothelial cell activation, platelet deposition, and tissue edema. Neuroinflammation plays an important role in many common neurodegenerative diseases [99]. Its accompanied by an increase of NO and/or O_2_^∙−^ with H_2_O_2_ production [100]. Generally, the inflammation is a protective process that protects the cells from detrimental agents, promoting tissue repair. In uncontrolled conditions the inflammatory process induces inordinate cell damage as it occurs in neurodegenerative disease. In particular, during neuroinflammation, microglia and astrocytes produce many inflammatory genes, including cytokines, chemokines, adhesion molecules, and proinflammatory transcription factors [101]. An increase of some transcription factors involved in inflammation was also found, such as NF-k*β*, peroxisome proliferator-activated receptor gamma (PPAR*γ*), and Sp1 in microglia cultures and AD brain [102–104]. Thus, the inflammatory mediators secreted by microglial and astrocytic cells contribute to neuronal dystrophy [105]. In these conditions microglia can produce ROS, NO, and proteolytic enzyme, enhancing the senile plaques and NFTs formation [106]. Furthermore, as a vicious cycle, the senile plaques induce the expression of proinflammatory cytokines and enzymes such as inducible NOS (iNOS) and cyclooxygenase enzyme (COX-2) in microglia cells, suggesting that all these factors can contribute to neurodegeneration [107].

In the case of AD many authors speculate that senile plaques and NFTs constitute the site of activation of a chronic inflammatory response. In fact, an interaction between A*β* peptide and CR3/Mac-1 (CD11b/CD18) on microglia has been observed. This interaction determines the activation of phosphatidylinositol 3-kinase (PI3K), which in turn phosphorylates p47^phox^, inducing the PHOX translocation and activation on microglia membrane increasing the production of O_2_^∙−^ and causing neuroinflammation [108, 109]. Thus the abnormal activation of microglia disrupts nerve terminals activity causing an alteration and a loss of synapses, which correlates with memory decline, leading to progression of AD [110]. Next to this, some studies have revealed an association between AD and mutations in different genes opening new strategies for comprehension of pathology [111, 112]. For example, genome exome and Sanger sequencing have revealed that heterozygous rare variants in triggering receptor expressed on myeloid cells 2 (TREM2) are associated with a significant increase in the risk of AD [113]. Also genome-wide investigations have revealed many polymorphisms in the human genome of AD patients. In particular, polymorphisms on clusterin (ApoJ, a potent regulator of complement induction) and CR1 (complement receptor) genes are genetically associated with sporadic AD [114, 115]. Moreover, the single nucleotide polymorphisms for cytokines and chemokines genes have been associated with AD risk [116].

In PD the activation of microglia has been amply demonstrated, suggesting an important role of neuroinflammation in the pathophysiology of PD. Activated microglia produce O_2_^∙−^ and NO, which in turn contribute to oxidative and nitrosative stress in the brain [117]. Notably, activated microglia and T lymphocytes, together with an increase of proinflammatory mediators, have been detected in the brain and cerebrospinal fluids of PD patients [118]. An increase of iNOS has been also revealed in activated microglia of PD subjects [118]. Moreover, the role of DA as being responsible for the ROS-mediated inflammation reaction in neurons was shown [119]. In fact, DA is stable in synaptic vesicles inside the cell; however once DA exists it is easily metabolized by MO. Alternatively, DA can undergo autooxidation determining the ROS production. As a result the microglia became active and produce proinflammatory cytokine, such as interleukin-1 (IL-1), tumor necrosis factor alpha (TNF-*α*) [120], and O_2_^∙−^ and NO [121], leading the generation of vicious cycle that further increases dopaminergic toxicity in the* substantia nigra*.

As for the other neurodegenerative diseases a characteristic of ALS pathology is the occurrence of a neuroinflammation, which activates microglia, astrocytes, and T-cells. In particular, the autopsy studies have demonstrated a microglia activation and an induction of activator transcription-3 (STAT3) in ALS spinal cord microglia [122]. Moreover, an upregulation of lipopolysaccharide/Toll Like Receptor 4 (LPS/TLR4) signaling associated genes has been observed in peripheral blood mononuclear cell (PMBCs) from ALS patients, suggesting chronic monocytes and macrophage activations [123]. Studies made on serum and cerebrospinal fluid (CSF) of 20 ALS patients show an increase of MCP1 and IL-8 levels, indicating a stimulation of proinflammatory cytokine cascade after microglia activation [124]. Also increased levels of IL-17, IL-6, and LPS are found in the serum of subjects with ALS [125]. ELISA assays have also demonstrated an increase of IL-15 and IL-12 in serum and CSF of 21 patients with ALS, suggesting that these molecules could be used as potential markers of immune activation in ALS [126]. Moreover, 2D gel electrophoresis analysis highlighted an increased activity of components of complement C3 in serum of ALS patients with respect to controls [127]. All these studies demonstrate the presence of an inflammatory and immune response in subjects with ALS.

## 4. Viral Infections and Neurodegeneration

**Figure 2:**
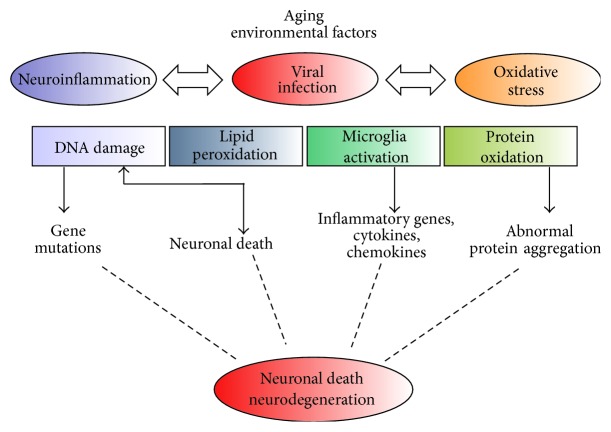
Different genetic and/or environmental factors lead to ROS increase during neurodegeneration. This accumulation triggered the activation of glia cells and the release of proinflammatory markers, stimulating thus a neuroinflammatory response. These events contribute to neuronal damage (DNA damage, lipid peroxidation, and protein oxidation) and axon degeneration, which ultimately caused neuronal death. In addition, virus infection can strengthen the ROS-mediated neurodegenerative signs in neurons and glia cells, producing functional and molecular hallmarks of neurodegeneration.

As mentioned above, a common feature of neurodegenerative disease is the chronic neuroinflammation and activation of microglia in the brains of patients with PD, AD, and ALS. In the last years, many studies show an association between virus infection and neurodegenerative as another important common feature of these disorders (Figure 2). In the second part of this review we will provide a detailed picture of how some virus infections can guide us to underpin mechanisms in neurodegeneration and amplify the damage mediated by oxidative stress.

Neuronal degeneration can be either directly or indirectly affected by viral infection. Viruses can injure neurons by direct killing, by cell lysis, and by inducing apoptosis. Different pathogens and/or their products may directly induce long-term degenerative effects, such as the deposit of misfolded protein aggregates, increased levels of oxidative stress, deficient autophagic processes, synaptopathies, and neuronal death. Viruses, bacteria, protozoa, and unconventional pathogens such as prion proteins have the ability to invade the CNS as described by De Chiara et al. (2012) [128]. There are different routes of entry of infectious agents into the CNS and they cause acute infections, which in some cases may be fatal or which may progress to become chronic illnesses [129, 130]. When the viruses enter into the nervous system, that is, they are neurotropic, it leads to activation of both innate and adaptive immune responses. Viral antigens preferentially activate the TLRs 3, 7, and 8 driving innate and adaptive immune responses and leading to neuronal damage, which occurs through direct damage, killing, release of free radicals, cellular activation, and inflammation, and induce a number of encephalopathies [58]. In particular, one of the secondary consequences of these encephalopathies can be the Parkinsonism that is both transient and permanent condition.

According to reviewed literature, and as discussed in depth below, a large number of studies demonstrate that the viruses are one of the main causes of degenerative diseases. In particular, as emerging from the review below, a growing interest is devoted to investigating the effects of H1N1 in PD (Section 4.1), of HSV1 in AD (Section 4.2), and of retroviruses in ALS (Section 4.3).

### 4.1. H1N1 in PD

In the last years, it has emerged that influenza virus has been implicated as a direct and an indirect cause of PD, although it was recently found that influenza can be considered as PD-like symptoms such as tremor, particularly in the month after an infection, but not with an increased risk of developing idiopathic PD [131].

Influenza virus is a respiratory pathogen contagious to humans, belonging to Orthomyxoviridae family, which are negative sense, single-stranded, segmented RNA viruses. In particular, a viral etiology for PD is based largely on epidemiological studies indicating a possible coincidence of PD with influenza flu pandemics, most notably the 1918-1919 “Spanish” influenza outbreak [132, 133]. In recent studies, Rohn and Catlin have shown the presence of influenza A virus within the* substantia nigra* pars compacta (SNpc) from postmortem PD brain sections [134]. They also identified colocalized influenza A and immune cells with caspase-cleaved Beclin-1 within the SNpc, which clearly indicated the role of neuroinflammation with influenza A virus's involvement in PD pathogenesis. Influenza A virus labelling was identified within neuromelanin granules as well as on tissue macrophages in the SNpc [134]. As mentioned above, the PD hallmark Lewy bodies are also composed mainly of aggregated *α*-synuclein. The formation of Lewy bodies is due to accumulation of normally produced Ser-129 phosphorylated *α*-synuclein [135]. It is demonstrated that H5N1 influenza virus progresses from the peripheral nervous system into the CNS and increases the phosphorylation and aggregation of *α*-synuclein [136]. Reviewed data suggest that influenza virus could have a role in the PD.

### 4.2. HSV1 in AD

Growing epidemiological and experimental evidence suggests that recurrent herpes simplex virus type-1 (HSV-1) infection is a risk factor for AD. It belongs to the family Herpesviridae, which is a large family of double-stranded DNA viruses. HSV-1 is a virus that primarily infects epithelial cells of oral and nasal mucosa [137]. The concept of a viral role in AD, specifically of HSV-1, was first proposed several decades ago [138, 139]. Several epidemiological studies have reported the presence of the HSV-1 genome in postmortem brain specimens from numerous AD patients, particularly those who carry the type 4 allele of the gene that encodes apolipoprotein E (APOE4), another potential risk factor for AD [140, 141]. Moreover, Wozniak et al. [142] have found the HSV-1 DNA in amyloid plaques of AD brains.

Several studies suggest that HSV-1 could be a possible major cause of amyloid plaques and hence possible aetiological factor in AD. Besides, genes related to HSV-1 reactivation have been detected in the brain of patients with familial AD, associated with *β*-amyloid deposits [143]. HSV-1 infection has also been shown to promote neurotoxic A*β* accumulation [144–146], tau phosphorylation [147], and cleavage [142]* in vitro*. Several studies have sought anti-HSV-1 IgM as well as IgG in serum from AD patients, showing that the risk of AD is increased in elderly subjects with positive titers of anti-HSV-1 IgM antibodies [148]. Genetic studies too have linked various pathways in AD with those occurring in HSV-1 infection [149].

The presented evidences suggest that HSV1 may have a critical role in AD pathogenesis.

### 4.3. Retroviruses in ALS

Retroviruses play an important role in the pathogenesis of ALS. In fact, several studies have reported retroviruses to be involved in ALS [150–154]. As found by [155], the reverse transcriptase (RT) enzyme of the retroviruses can convert RNA into complementary DNA. The first demonstration of retroviral involvement in ALS dates back to 1975 when Viola et al. [156] found RT activity in cytoplasmic particulate fraction from two Guamanian ALS but not in brains from two control individuals. At that time, a growing interest was in finding the retroviral.

Other studies showed that the RT is present more frequently in ALS patients' sera compared to that of control and the levels of the activity in ALS patients were comparable to that in HIV-infected patients [157, 158].

ALS-like syndromes are developed in a small percentage of persons infected with the human immunodeficiency virus-1 (HIV-1) or human T-cell leukemia virus-1 (HTLV-1). HIV-infected patients may develop neurological manifestations that resemble classical ALS although it occurs at a younger age and they may show a dramatic improvement following the initiation of antiretroviral therapy. On the other hand, HTLV-1 associated ALS-like syndrome has several features that may distinguish it from classical ALS. However, most patients with probable or definite ALS show no evidence of HIV-1 or HTLV-1 infection [159]. Moreover, studies have shown increased HERV-K expression in both serum and brain tissue in ALS patients [160]. Furthermore, in a recent study it has been shown that HERV-K is activated in a subpopulation of patients with ALS and that its envelope protein may contribute to neurodegeneration [161]. These evidences suggest that retroviruses are involved in the pathophysiology of ALS.

## 5. Viral Infections and Oxidative Stress in Neurodegenerative Disease

Frequently viral infections cause changes in the redox state in host cells [162–166]. Many viral infections can cause an increased generation of ROS and RNS, which can be caused by both direct effects of virus on cells and inflammatory responses of the chronic viral host. In the presence of surplus ROS, the pathogen-mediated proteins can induce pathologic changes in neural tissue and lead to chronic inflammation of the brain, as seen in classical neurodegenerative diseases.

### 5.1. HSV1

HSV1-1 when infecting neurons and glia cells induce the production of proinflammatory cytokines produced by microglia and infiltrating macrophages, as well as the production of chemokines and antiviral cytokines [167, 168]. Several studies have shown that during HSV-1 infection into the cell a depletion of GSH, the production of ROS, the induction of mitochondrial DNA damage, and endoplasmatic reticulum stress with consequent alteration of the intracellular redox state towards a prooxidant state occur [166, 169–171].

More data indicate that virus infection induced oxidative damage in the brain. In particular, Schachtele et al. (2010) [172] have shown that HSV-1 induced neural cell oxidative tissue damage and cytotoxicity, which are mediated by microglial cell through a TLR2-dependent mechanism. In other studies increases in ROS levels, lipid peroxidation, and protein nitrosylation were reported when there is HSV-1 infection [167, 173, 174]. Furthermore, in the recent study Santana et al. (2013) have shown that oxidative stress enhances the accumulation of intracellular A*β* and the inhibition of A*β* secretion induced by HSV-1 infection [175]. Several studies suggest that HSV-1 induced oxidative stress in neuronal cells may trigger *β*- and *γ*-secretase activation and, consequently, APP processing and A*β* formation. These findings demonstrate that HSV-1 infection of neuronal cells can generate multiple APP fragments with well-documented neurotoxic potentials [147].

### 5.2. Influenza Virus

*Influenza *virus uses host cell structures and metabolic pathways for its life-cycle. In particular, intracellular redox state changes, for example, GSH depletion or ROS or RNS increase, have been detected during influenza virus infection [165]. On the other hand, it has been recently demonstrated that NOX4 enzyme, the main source of ROS production during influenza virus infection, regulates specific steps of virus life-cycle [34]. Virus-induced GSH decrease is pivotal for viral replication by allowing the folding and maturation of viral hemagglutinin [176] and activating cellular kinases involved in nucleocytoplasmic traffic of viral proteins [177].

On the basis of these evidences it can be assumed that the infection of influenza virus amplifies the effects of oxidative stress, which contribute to neuronal damage.

### 5.3. Retroviruses

Garaci et al. [178] demonstrated that* in vitro* HIV infection significantly decreases the GSH content of human macrophages. In addition, recent work has shown that HIV-1 induces ROS production in astrocytes and microglia [179, 180]. Dasuri et al. [180] have shown that oxidative stress is involved in the pathology of HIV-associated neurocognitive disorders. HIV-infected monocytes and T-cell, to enter in the cell, use the glycoprotein gp120. The viral protein gp120 can directly induce apoptosis in neurons and increase oxidative stress through GSH and lipid peroxidation [179].

The increases of ROS plays a role in viral pathogenesis probably because the increase of oxidative stress, generated when viruses infect the aged neuronal cells, may contribute to increasing the production of misfolded proteins and hence to the pathogenesis of neurodegenerative diseases.

Data discussed in this review suggest that viruses can be causative agents or, at least, cofactors of some neurodegenerative diseases. Therefore, much attention should be paid to infectious and, especially, viral agents among the environmental factors contributing to neurodegenerative diseases.

## 6. Conclusions

Although numerous studies have been made to understand the genetic/molecular mechanisms that underly the different neurodegenerative diseases, the comprehension of how redox imbalance is implicated in viral infection during neuronal damage is still unclear. In particular, understanding whether the redox imbalance is the cause or the effect of an increased propensity of brain cells to infection would be of great importance to develop new therapeutic strategies to target redox/inflammatory markers in brain inflammation and neurodegenerative disorders.

## Abbreviations

MPTP: 1-Methyl-4-phenyl-1,2,3,6-tetrahydropyridine
MPP+: 1-Methyl-4-phenylpyridinium
HNE: 4-Hydroxynonenal
8-OHG: 8-Hydroxyguanosine
AP-1: Activator protein-1
STAT3: Activator transcription-3
AGE: Advanced glycation end product
AD: Alzheimer's disease
APP: Amyloid precursor protein
ALS: Amyotrophic lateral sclerosis
A*β*: *β*-Amyloid peptide
CNS: Central Nervous System
CSF: Cerebrospinal fluid
CR1: Complement receptor
COX-2: Cyclooxygenase enzyme
DAT: DA transporter
DOPAC: Dihydroxyphenylacetic acid
DA: Dopamine
ETC: Electron transport chain
eNOS: Endothelial nitric oxide synthase
GSH: Glutathione
HO-1: Heme Oxygenase-1
HSV-1: Herpes simplex virus type-1
H_2_O_2_: Hydrogen peroxide
^∙^OH: Hydroxyl radical
IL-12: Interleukin-12
IL-15: Interleukin-15
IL-17: Interleukin-17
IL-1*β*: Interleukin-1*β*
IL-6: Interleukin-6
IL-8: Interleukin-8
iNOS: Inducible nitric oxide synthase
INF-*γ*: Interferon-*γ*
LPS: Lipopolysaccharide
MIP1*α*: Macrophage inflammatory protein 1*α*
MIP1*β*: Macrophage inflammatory protein 1*β*
NFM, NFH: Medium and high molecular weight neurofilaments
mtGSH: Mitochondrial glutathione
MO B: Monoamine oxidase B
MCP1: Monocyte chemotactic protein 1
NOX: NADPH oxidase
NFTs: Neurofibrillary tangles
NO: Nitric oxide
NF-k*β*: Nuclear factor-k*β*
PD: Parkinson's disease
PPAR*γ*: Peroxisome proliferator-activated receptor gamma
ONOO^−^: Peroxynitrite
PMBCs: Peripheral blood mononuclear cells
RNS: Reactive nitrogen species
ROS: Reactive oxygen species
SNpc: * Substantia nigra* pars compacta
O_2_^∙−^: Superoxide anion
SOD2: Superoxide dismutase 2
TLR: Toll Like Receptor
TNF-*α*: Tumor necrosis factor-*α*
TREM2: Triggering receptor expressed on myeloid cells 2
TH: Tyrosine hydroxylase.
